# Implementation of substance use screening in rural federally-qualified health center clinics identified high rates of unhealthy alcohol and cannabis use among adult primary care patients

**DOI:** 10.1186/s13722-023-00404-y

**Published:** 2023-09-20

**Authors:** Jennifer McNeely, Bethany McLeman, Trip Gardner, Noah Nesin, Vijay Amarendran, Sarah Farkas, Aimee Wahle, Seth Pitts, Margaret Kline, Jacquie King, Carmen Rosa, Lisa Marsch, John Rotrosen, Leah Hamilton

**Affiliations:** 1 Department of Population Health, Section on Tobacco, Alcohol and Drug Use, New York University Grossman School of Medicine, 180 Madison Ave., 17th Floor, New York, NY 10016 USA; 2 Center for Technology and Behavioral Health, Geisel School of Medicine at Dartmouth College, 46 Centerra Parkway, Evergreen Center, Suite 315, Lebanon, NH 03766 USA; 3 Penobscot Community Health Care (PCHC), 103 Maine Avenue, Bangor, ME 04401 USA; 4 Department of Psychiatry, New York University Grossman School of Medicine, 1 Park Ave, New York, NY 10016 USA; 5 The Emmes Company, 401 N. Washington St., Rockville, MD 20850 USA; 6 National Institute on Drug Abuse, c/o NIH Mail Center, NIDA 3@FN MSC 6022, 16071 Industrial Drive—Dock 11, Gaithersburg, MD 20892 USA; 7 Kaiser Permanente Washington Health Research Institute, 1730 Minor Avenue, Seattle, WA 98101 USA

**Keywords:** Substance use disorders, Alcohol, Drug use, Opioid use disorder, Screening, Primary care, Implementation, Rural

## Abstract

**Background:**

Screening for substance use in rural primary care clinics faces unique challenges due to limited resources, high patient volumes, and multiple demands on providers. To explore the potential for electronic health record (EHR)-integrated screening in this context, we conducted an implementation feasibility study with a rural federally-qualified health center (FQHC) in Maine. This was an ancillary study to a NIDA Clinical Trials Network study of screening in urban primary care clinics (CTN-0062).

**Methods:**

Researchers worked with stakeholders from three FQHC clinics to define and implement their optimal screening approach. Clinics used the Tobacco, Alcohol, Prescription Medication, and Other Substance (TAPS) Tool, completed on tablet computers in the waiting room, and results were immediately recorded in the EHR. Adult patients presenting for annual preventive care visits, but not those with other visit types, were eligible for screening. Data were analyzed for the first 12 months following implementation at each clinic to assess screening rates and prevalence of reported unhealthy substance use, and documentation of counseling using an EHR-integrated clinical decision support tool, for patients screening positive for moderate-high risk alcohol or drug use.

**Results:**

Screening was completed by 3749 patients, representing 93.4% of those with screening-eligible annual preventive care visits, and 18.5% of adult patients presenting for any type of primary care visit. Screening was self-administered in 92.9% of cases. The prevalence of moderate-high risk substance use detected on screening was 14.6% for tobacco, 30.4% for alcohol, 10.8% for cannabis, 0.3% for illicit drugs, and 0.6% for non-medical use of prescription drugs. Brief substance use counseling was documented for 17.4% of patients with any moderate-high risk alcohol or drug use.

**Conclusions:**

Self-administered EHR-integrated screening was feasible to implement, and detected substantial alcohol, cannabis, and tobacco use in rural FQHC clinics. Counseling was documented for a minority of patients with moderate-high risk use, possibly indicating a need for better support of primary care providers in addressing substance use. There is potential to broaden the reach of screening by offering it at routine medical visits rather than restricting to annual preventive care visits, within these and other rural primary care clinics.

## Introduction

Rural communities are highly impacted by unhealthy alcohol and drug use. In the United States and globally, the prevalence of alcohol use disorder and alcohol-related harms are greater in rural areas than in urban or suburban areas [1, 2]. Rural communities have faced disproportionate rates of overdose death, and remain affected during the current ‘Fourth Wave’ of the opioid crisis, with elevated rates of opioid and psychostimulant use, often co-occurring with mental health disorders [3–5]. Cannabis use has also risen across the US, including in rural areas, in recent years, and frequent cannabis use is somewhat more prevalent outside of metropolitan areas [6, 7]. Reducing the health impact of drug use is challenging in rural areas, which have limited access to treatment and harm reduction services, and unique barriers to reaching and engaging people who use substances [3, 8–10].

Health care visits represent an opportunity to identify unhealthy use and provide interventions, and primary care settings are especially important in rural areas, where patients have less access to acute and specialty care [11]. Screening adult primary care patients for unhealthy alcohol use has been recommended by the United States Preventive Services Task Force (USPSTF) since 1996 [12], and drug screening since 2020 [13]. Although screening for substance use in medical settings could be an important step toward reducing the negative health impacts of alcohol and drugs, it is infrequently done [14–18].

To address the need to expand substance use screening in primary care, we conducted a multi-site NIDA Clinical Trials Network study on the feasibility of implementing electronic health record (EHR)-integrated drug and alcohol screening [19]. Although this study found overall support for integrating validated screening instruments into routine primary care visits, it was limited to clinics located in large urban centers [19]. Given the unique needs of rural health systems, an ancillary study was subsequently undertaken to explore whether the same model of EHR-integrated screening and assessment for substance use used in the parent study could translate to rural primary care.

Rural clinics face unique barriers to implementing substance use screening, including reduced availability of behavioral health and substance use disorder (SUD) treatment, workforce shortages, and patients with more complex physical and mental health needs, and more severe poverty, than is typically seen in urban settings [3, 11, 20, 21]. In early focus groups conducted for this study, providers and patients identified barriers including concerns about stigma, EHR privacy, staff comfort/training with respect to substance use discussion and treatment, and having sufficient time to conduct screening [22]. While many of these barriers are commonly encountered even in urban clinics [23], issues related to privacy in small communities, as well as difficulty hiring staff who are knowledgeable about substance use, are particularly powerful in rural settings [22, 24].

To examine the feasibility of implementing EHR-integrated screening in this context, a rural Federally Qualified Health Center (FQHC) in Northern Maine with multiple clinical sites was identified to participate in this ancillary study. FQHCs are key health care providers in rural communities [25], and could be a favorable environment for addressing substance use because many are already offering integrated behavioral health services. As in the parent study, the overarching goal of this ancillary study was to facilitate the implementation of substance use screening approaches that would optimize the screening rate and had good potential for sustainability. This article presents implementation outcomes from the first year of screening in the three participating FQHC clinics.

## Methods

### Study design

This ancillary study was designed with the same elements as its parent study, which was previously described [19]. Implementation was guided by the Knowledge to Action (KTA) framework, which can inform the selection and implementation of new clinical practices [26, 27]. Study activities focused on the KTA ‘action cycle,’ and consisted of adapting, implementing, and evaluating the use of substance use screening tools with implementation outcome measures. Although the implementation process in these ancillary study clinics was similar to that of the parent study, all of the study activities described herein were conducted at the Maine FQHC clinic sites. To inform the screening implementation approach, barriers were assessed through focus groups and interviews with stakeholders [22], and the EHR tools developed to support screening were tailored through multiple rounds of usability testing conducted with providers and staff at the three participating clinics.

The screening rollout was intentionally staggered, with Clinic 1 initiating first (November 2018), followed by the remaining clinics in spring 2019 (Clinic 2 in April 2019, Clinic 3 in May 2019). This schedule allowed for technical and logistical issues to be addressed in the first clinic, prior to rolling out the program to additional clinics. Following the initiation of screening at each clinic, another round of focus groups with clinic providers was conducted to understand issues related to early implementation [24].

Implementation outcomes were assessed for 12 months following initiation of screening, using EHR data gathered at the clinic level. Data collection ended in May 2020. Our primary outcome was adoption of screening, measured as the screening rate for alcohol and drug use. Secondary outcomes were the prevalence of unhealthy substance use detected via screening, and provider adoption of the EHR-integrated clinical decision support tool. The Institutional Review Boards at New York University Grossman School of Medicine and Dartmouth College approved the study. Standards for Quality Improvement Reporting Excellence (SQUIRE) reporting guidelines for healthcare quality improvement studies were followed [28].

### Setting

The study was conducted in three clinics of a single rural FQHC located near Bangor, Maine. Recreational cannabis was legalized in Maine prior to the start of the study, in late 2016. Medicaid expansion was implemented in Maine in January 2019, which was during the study period. The participating FQHC is the largest in the state, with a total of nine primary care clinics (eight with adult patients, one pediatrics only) serving approximately 60,000 patients a year.

The three study clinics were chosen by the FQHC based on their geographic proximity to one another, size (medium-large clinics were preferred), use of a shared EHR system, and availability of an on-site clinical champion. Patients served by these FQHC clinics include rural, poor, unhoused, and medically underserved populations. Prior to the study, none of the participating clinics were systematically screening patients for alcohol or drug use. Table 1 identifies key characteristics of the study clinics. Primary care providers (PCPs) were physicians (MD and DO), nurse practitioners (NP), and physician assistants (PA). All clinics had integrated behavioral health services that primarily focused on mental health care (not substance use), and offered office-based buprenorphine treatment for opioid use disorder. All clinics used a Centricity™ EHR (GE Healthcare, Chicago, Illinois).

**Table 1:** Clinic characteristics

	Clinic 1	Clinic 2	Clinic 3
Primary care providers			
MD or DO	4	7	5
NP or PA	9	5	6
Patient census (approximate)	5900	9200	6300
On-site behavioral health	Yes	Yes	Yes
On-site buprenorphine treatment for opioid use disorder	Yes	Yes	Yes

### Screening program elements

Clinics used screening program elements similar to those of the parent study, which have been demonstrated to increase adoption of screening and interventions by health care providers [29–32]. These elements are summarized in Table 2 and include an EHR-integrated clinical decision support system (CDSS) consisting of a clinical reminder and counseling script, and use of a validated screening tool. The approach to delivering these elements of the screening program, including the visit types that were eligible for screening, frequency of clinical reminders, and content of the counseling script, were based on what was learned in the focus groups and through usability testing. A notable difference from the parent study was the screening tool used by the clinics. The parent study used the single-item screening questions for alcohol and drugs, followed by AUDIT-C and/or DAST-10 [19], whereas the ancillary study clinics agreed to use the Tobacco, Alcohol, Prescription Medication and Other Substance Use (TAPS) Tool [33, 34]. The TAPS Tool is a 2-part screener and brief assessment, the structure and scoring of which were previously reported [33, 35]. TAPS-1 identifies any past-year use of tobacco, alcohol (exceeding daily limit of 5 drinks for men or 4 drinks for women), prescription medications (non-medical use), or illicit drugs (including cannabis). TAPS-2 is administered for each substance class endorsed in TAPS-1, and identifies current (past 3 months) use and risk level for up to 9 types of substances: tobacco, alcohol (above daily limits), cannabis, opioids, illicit stimulants (cocaine, methamphetamine), heroin, and non-medical use of prescription sedatives, opioids and stimulants. Responses are summed within each substance type to generate a substance-specific risk score, ranging from 0 to 3 for tobacco and other drugs, and 0–4 for alcohol. TAPS score of 0 indicates no current use of that substance, a score of 1 indicates moderate risk use (called ‘problem use’ in the TAPS validation study), and a score of 2 indicates higher risk use [33]. For example, an individual who reports at least 1 day of alcohol use above the recommended daily limit on TAPS-1 would be administered the TAPS-2 alcohol items. If they reported on the TAPS-2 no alcohol use above the daily limits in the past 3 months they receive a score of 0 for alcohol; with any use above the daily limits their score is 1; and use above the daily limits plus one problem related to alcohol generates a substance specific alcohol score of 2.

**Table 2:** Screening program elements and implementation strategy utilized by study clinics

Element	Description
**Screening Program Elements**	
EHR	Centricity EHR
	Custom software to collect screening on tablets and transfer into EHR
Visits eligible for screening	Annual adult preventive care appointments
Screening tools	Tobacco, alcohol, prescription medication, and other substance (TAPS) Tool^a^
	TAPS-1 for screening (any past year use)
	TAPS-2 for brief assessment (current use, risk level)
	Established cutoffs categorized level of risk: 0 = low risk/no current use; 1 = moderate risk; 2+ = high risk
Mode of screening	Self-administered on a tablet while in waiting room
	Completed by medical assistant for patients needing assistance
CDSS: clinical reminder	Alert in EHR indicating that a patient is due for screening (based on age, visit type and not being screened in the past 12 months)
CDSS: counseling and referrals	Template built into the EHR provided guidance for conducting and documenting a brief intervention
	Accessed in the TAPS results page; tab with fillable fields to document patient responses
	Designed to be delivered in ~ 5 min
	Guided providers through the four major components of a brief negotiated interview: raising the subject, providing feedback, enhancing motivation, and formulating a plan^b^
	Order set for patient education materials and referral for treatment or social work assessment
**Implementation strategy**	
Clinical champions	Each clinic had one ‘clinical champion’ PCP
	Worked with the research team and led implementation at their clinic
	Met approximately monthly with the practice facilitator
	Assisted with training of clinic staff
Practice facilitation	One staff member from the FQHC’s quality improvement unit for reporting, monitoring and ongoing education
	Assisted by one trained research assistant who worked across all clinics
Training	Conducted by FQHC substance use clinical leader (TG)
	Offered during established meeting times to facilitate attendance
	PCPs: One group training session on screening, brief intervention, and use of the CDSS (30–45 min)
	Medical assistants and front desk staff: one brief training focused on the screening workflow
	PCPs and medical assistants who were unable to attend group training had the option of receiving individual training

^a^See ref. [33]

^b^See ref. [64]

The clinics chose to screen for substance use only during adult annual preventive care visits, which all adult primary care patients are intended to complete once per year. They opted for patient self-administered screening on tablet computers, conducted in the waiting room prior to the PCP visit, as the predominant screening approach. Tablets were managed by clinical staff, and the clinics were responsible for maintaining and operating them. Because the Centricity^™^ EHR did not accommodate patient-facing questionnaires, the clinics contracted with an outside software development company (PatientLink© [PatientLink Enterprises, Oklahoma City, OK]) to build the tablet-based screener that would be used by patients. The software allowed patients to complete the TAPS Tool as well as depression screening (Patient Health Questionnaire (PHQ)-2/9) on the tablet, with results seamlessly uploaded into the EHR at the point of care. TAPS Tool screening results were visible to the PCP in a study-designed clinical decision support system (CDSS) integrated into the EHR. To access the CDSS, providers clicked on a tab labeled ‘TAPS’, located on the home screen just beneath the tab for viewing vital signs. The CDSS landing page presented a summary of the screening results, from which providers could click on an ‘intervention’ tab that provided guidance for conducting and documenting a brief motivational counseling intervention, an option for selecting patient education handouts, and an order set for treatment referrals. Provider use of the CDSS was recommended for patients with moderate or high risk use of alcohol and/or drugs.

### Implementation strategy

All clinics used the same implementation strategy, elements of which are summarized in Table 2.

Clinical champions: Clinical champions can be integral to enacting practice change, by supporting, marketing, and overcoming barriers to implementation [36, 37]. In our study, each clinic had a clinical champion who was a practicing PCP at their site. Champions facilitated the integration of screening into local workflows, provided ongoing support and education to the clinical staff, identified problems related to adoption and workflow, and met approximately once/month with the local research team to monitor implementation.

Practice facilitation: Practice facilitation is an implementation strategy wherein trained facilitators work with clinic leaders and staff to implement evidence-based practices [38–40]. Practice facilitation was led by one staff member from the FQHC’s quality improvement unit, who was a former medical assistant in one of the study clinics, and assisted by a research assistant. All clinics received practice facilitation for approximately 3 months prior to the start of the screening program, and on an ongoing basis throughout the first year.

Training: Training was led by a psychiatrist from the FQHC (TG or VA) who was a clinical leader of substance use services. All clinical staff received a one-hour group training session that covered screening and brief intervention for unhealthy substance use, and use of the EHR-integrated screening tools. Additional training for medical assistants and front desk staff was done by the practice facilitator. Medical assistants were trained on administering the screening instrument to patients who needed assistance and accepting results from the tablets into the EHR. Front desk staff were trained on using the clinical reminder, asking patients to complete screening prior to the visit, and on distribution and collection of the tablets.

### Implementation outcome measures

Implementation data were extracted from the EHR for the first 12 months of screening at each clinic. Summary reports of the tracked implementation outcomes were generated weekly for the first 3 months of screening at each clinic, and then quarterly. Reports were sent to the investigators and then reviewed by the practice facilitator, clinical champion, and research team at each site to inform ongoing implementation. Implementation reports allowed the research team and practice facilitators to identify unanticipated technical or logistical issues arising in the implementation process (e.g. data extraction codes missing information on a specific substance, or workflow issues leading to sudden drops in screening rate at a specific clinic). Each data extract included the (1) number of patients eligible for screening (adults 18 years or older, who had an annual preventive care visit in the given time period), (2) number of patients screened, (3) TAPS Tool results indicating the prevalence of moderate and high risk use of tobacco, alcohol, cannabis, other illicit drugs, and prescription medications, and (4) provider use of the CDSS counseling script.

### Analysis

The FQHC sent EHR data extracts capturing screening implementation outcomes to the study’s data and statistical coordinating center (The Emmes Company) throughout the first 12 months. Demographic characteristics were extracted after the end of the study period. Descriptive statistics were calculated to characterize the rates and frequencies of the pre-defined implementation outcomes and patient demographics. Screening rate was calculated as the proportion of eligible patients presenting for an annual preventive care visit who completed the TAPS-1 screener. Prevalence of moderate and high risk use was calculated based on the TAPS score, using standard cutoffs (1 + for moderate risk, 2 + for high risk). Because the counseling script was only suggested when patients screened positive for moderate or high risk use of alcohol and/or drugs, the rate of counseling was calculated based on the moderate-high risk population. All analyses were performed using SAS version 9.4.

## Results

Demographic characteristics of patients eligible for screening are shown in Table 3. Patient characteristics were similar across the 3 clinics. On average, patients were in their mid-40s, were predominantly white and female, and had private insurance. Characteristics of the screening-eligible patients were similar to those of the general patient population of these 3 clinics, except patients in the study sample had somewhat higher rates of private insurance (63–66% vs. 42–45%) and lower rates of Medicaid coverage (11–16% vs. 19–26%).

**Table 3:** Demographic characteristics of patients eligible for screening at each clinic

Characteristic	Clinic 1	Clinic 2	Clinic 3
Total number of eligible patients	1641^	1429	942
Age			
Mean	46.7	47.7	49.1
SD	14.85	15.66	15.49
Sex			
Female	1071 (65.3%)	846 (59.2%)	561 (59.6%)
Male	570 (34.7%)	583 (40.8%)	381 (40.4%)
Race*			
Black/African American	16 (1.0%)	7 (0.5%)	2 (0.2%)
White	1507 (91.8%)	1316 (92.1%)	913 (96.9%)
Asian	20 (1.2%)	14 (1.0%)	6 (0.6%)
American Indian/Alaska Native	15 (0.9%)	5 (0.3%)	7 (0.7%)
Other	10 (0.6%)	13 (0.9%)	2 (0.2%)
Multiracial	16 (1.0%)	2 (0.1%)	1 (0.1%)
Missing	57 (3.5%)	72 (5.0%)	11 (1.2%)
Health insurance			
Medicaid	268 (16.3%)	159 (11.1%)	113 (12.0%)
Medicare	178 (10.8%)	231 (16.2%)	152 (16.1%)
Private insurance	1073 (65.4%)	901 (63.1%)	622 (66.0%)
No insurance	67 (4.1%)	78 (5.5%)	29 (3.1%)
Other	55 (3.4%)	60 (4.2%)	26 (2.8%)

^3 patients under 18 years of age were removed

^*^No ethnicity data was collected within the health system

Table 4 presents the screening implementation outcomes, which were similar across the 3 clinics. A total of 20,300 adult patients visited the three study clinics during the period, of which 4015 (19.8%) had a screening-eligible visit. Screening was completed by 3749 patients, representing 93.4% of those eligible for screening, and 18.5% of all adult patients with any primary care visits during the study period. Screening was predominantly self-administered, with 92.9% of screens completed by patients on tablets.

**Table 4:** Screening implementation outcomes for the first 12 months of screening

	Clinic 1 *N* (%)	Clinic 2 *N* (%)	Clinic 3 *N* (%)	Total N (%)
Total patients with visits	6861	9477	3962	20,300 (100.0%)
Patients with eligible visits^a^ [N (% of total patients)]	1644^b^ (24.0%)	1429 (15.1%)	942 (23.8%)	4015 (19.8%)
Screening completed (N)	1522	1319	878	3749
Screening rate among patients with eligible visits (%)	92.6%	92.3%	93.2%	93.4%
Screening rate among patients with any visit (%)	22.6%	13.9%	22.2%	18.5%
Self-administered screening [N (% of those screened)]	1443 (93.0%)	1221 (92.6%)	818 (93.2%)	3482 (92.9%)
Moderate-high risk for tobacco^c^	261 (16.8%)	156 (11.8%)	131 (14.9%)	548 (14.6%)
Moderate risk	84 (5.4%)	61 (4.6%)	37 (4.2%)	
High risk	177 (11.4%)	120 (9.1%)	94 (10.7%)	
Moderate-high risk for alcohol^c^	495 (31.9%)	379 (28.7%)	265 (30.2%)	1139 (30.4%)
Moderate risk	324 (20.9%)	290 (22.0%)	146 (16.6%)	
High risk	171 (11.0%)	170 (12.9%)	119 (13.6%)	
Moderate-high risk for cannabis^c^	174 (11.2%)	130 (9.9%)	102 (11.6%)	406 (10.8%)
Moderate risk	105 (6.8%)	103 (7.8%)	57 (6.5%)	
High risk	69 (4.4%)	51 (3.9%)	45 (5.1%)	
Moderate-high risk for illicit drugs^c,d^	–	–	–	12 (0.3%)
Moderate-high risk for prescription drugs^c,d^	–	–	–	23 (0.6%)
Counseling documented^e^	88/569 (15.5%)	94/520 (18.1%)	61/304 (20.1%)	243/1393 (17.4%)

^a^Health system selected annual preventive care visits as the eligible visit type

^b^At Clinic 1, there were 3 patients under age 18 who had annual preventive care visits

^c^Risk levels were calculated as a percentage of patients screened

^d^Moderate- and high-risk use of illicit drugs and prescription drugs had cell numbers below 10, so only summary results are presented

^**e**^Counseling was captured if documented in the EHR counseling template and is calculated as the number of patients provided counseling out of all patients screening at moderate to high risk for alcohol or any drug(s) on the TAPS Tool

Among screened patients, 14.6% screened positive for moderate-high risk tobacco use, 30.4% screened positive for moderate-high risk alcohol use, and 10.8% screened positive for moderate-high risk cannabis use. A total of 0.3% of the screened patients had moderate-high risk use of illicit drugs (not including cannabis), and 0.6% had moderate-high risk use of prescription drugs. Counseling was documented in the CDSS tool for 17.4% (range 15.5–20.1%) of patients who screened positive for moderate-high risk alcohol or drug use.

## Discussion

Implementation of the EHR-integrated screening program in three rural FQHC clinics resulted in 3749 patients (93.4% of those with screening-eligible annual preventive care visits) receiving alcohol and drug screening in the first year. This ancillary study demonstrates the feasibility of incorporating screening into routine primary care in rural settings, and attests to the potential for screening to identify high rates of clinically significant alcohol and drug use. Successful implementation of screening aligns these clinics with the current USPSTF guidelines for alcohol and drug screening in adult primary care patients [12, 13].

The decision to implement screening only during annual preventive care visits was informed by the pre-implementation focus groups, in which there was concern about overburdening clinical staff by screening at all visit types, and by an earlier pilot program in the FQHC that was not well received. While annual visits may be a natural context for screening for substance use, since they are typically allotted more time and less driven by acute complaints, our findings reveal the limitations to this approach. Although the clinics were highly successful in screening patients who presented for annual visits (93.4% screening rate), they screened less than 20% of all adult patients who had primary care visits during the study period. This is similar to what was observed in the parent study, wherein the clinics that targeted any visit types were able to screen over 90% of patients with any visit during the implementation period, while those clinics that screened only at annual visits had lower screening rates, ranging from 24 to 72% [19]. Many patients simply do not keep appointments for annual preventive care visits, and are never screened as a result. A prior study found that publicly insured patients, as well as racial/ethnic minorities, have higher rates of missed appointments [41]. Targeting specific scheduled visit types may disproportionately impact patients with weaker connections to primary care, who may also be at elevated risk for unhealthy substance use.

Approximately 10% of patients screened positive for moderate-high risk cannabis use, while less than 1% screened positive for moderate-high risk use of illicit or prescription drugs. This rate of drug use is more than 10 times that detected in the parent study clinics, where 0.8% of patients screened positive for moderate-high risk use of any drug (including cannabis) [19]. While the context of screening implementation was different in these ancillary study clinics—most notably that recreational cannabis was legalized in Maine years prior to the launch of our study, but was legal in only one of the parent study states at the time of the study—the actual rates of unhealthy drug use were not anticipated to differ so greatly. The higher reporting of drug use could potentially be influenced by the use of a screening tool (TAPS Tool) that allowed patients to report cannabis use specifically, while the screeners in the parent study (single-item screening question for drugs, followed by DAST-10) asked about all drug use without discriminating between drug classes.

Having a screener that distinguishes cannabis from other drugs may be important given the increasing prevalence of cannabis use in the U.S., and particularly in cannabis-legal states. A previous study in a private health system located in the cannabis-legal state of Washington similarly found that asking about cannabis use separately from other drug use resulted in increased identification of cannabis use [42]. Patients who use cannabis for medical purposes or live in a state where recreational use is legal may perceive cannabis use as being akin to tobacco or alcohol use—i.e., carrying some health risks, but not of the same severity as use of other drugs. For these patients, screening instruments that ask about cannabis as a subset of all drug use can seem inappropriate or stigmatizing, and a cannabis-specific screener could facilitate more accurate reporting [43, 44].

We cannot discern from this study the degree to which the fairly low rate of screening-detected unhealthy illicit or prescription drug use is an accurate reflection of the true prevalence in this patient population, versus being falsely low due to patients being unwilling to disclose their use of these drugs. The National Survey on Drug Use and Health (NSDUH) also indicates that prevalence of drugs other than cannabis is relatively low in the general adult population, but higher than the rates detected on screening in our study. In 2019, which was the year of screening implementation at the study clinics, NSDUH reported prevalence of past-year use of illicit drugs among adults age 26 or older ranging from 0.3% (heroin) to 1.7% (cocaine), and misuse of prescription drugs ranging from 1.2% (stimulants) to 3.4% (opioids), while the rate of past-year cannabis use was substantially higher at 15.2% [45]. While these results for the general US population may not generalize to the region of rural Maine where the study was conducted, they suggest that there may be some underreporting of drug use. During the earlier focus groups conducted at these clinics, patients discussed reluctance to disclose drug use due to stigma, privacy concerns, and fear of the consequences for their medical care, concerns that may be heightened when they know that results are integrated into the EHR and visible to their medical providers [22].

The use of a predominantly self-administered screening approach may have contributed to higher reporting of alcohol and drug use. Prior research on self-administered screening indicates that it achieves more accurate reporting of stigmatized conditions than does in-person screening [19, 46–48]. While there was initially some skepticism among clinic staff about using electronic tablets to administer screening in a rural population, where internet access and digital health literacy may be limited, we found that it was feasible and well accepted by patients. Although patients had the option of completing screening with the medical assistant, 92.9% of screening was self-administered on the tablets. Findings from our early focus groups and ongoing anecdotal reports from the front desk staff and medical assistants at the clinics further support its acceptability [24].

The CDSS was used to document counseling in 17.4% of patients with moderate-high risk alcohol or drug use. While this is higher than the rates of counseling in the parent study clinics (where counseling ranged from 0.1 to 12.5%), there is room for improvement. Although it is possible that PCPs discussed substance use but did not document it in the CDSS counseling script, prior research indicates that counseling may not have been adopted because of the time required (at least 5 min), along with provider discomfort and lack of knowledge about substance use that may have inhibited them from engaging patients in discussion of these behaviors [49–52]. While these FQHC clinics had on-site behavioral health providers, they were primarily focused on mental health care, and there were not dedicated programs for alcohol and cannabis treatment. A perception by primary care providers and patients that treatment services were lacking may have discouraged them from discussing substance use. Training of PCPs on screening and interventions for substance use was limited, and it is possible that more intensive training would have led to more counseling and referrals. However, prior research indicates that passive education alone is generally insufficient to change provider behavior, though one-on-one training (such as academic detailing) could have greater effects [53, 54]. Team-based approaches, including collaborative care models with a focus on population management and delivery of evidence-based treatment for substance use, could help PCPs to provide interventions for unhealthy alcohol and drug use [55–57], but are not offered in most primary care settings.

### Limitations

Our study has limitations stemming from its origins as an implementation feasibility study. Clinics were not randomized, and so it is not known to what extent the screening rates observed during the study period were attributable to the screening program versus reflecting an evolution of practice that may have occurred even without these implementation efforts. Patients with screening-eligible visits during the implementation year were more likely to have private insurance than in the general clinic population, suggesting some limitations in generalizing these findings to all patients within the health system. Maine’s Medicaid expansion occurred during the study, which could have introduced changes in primary care utilization that were not anticipated. All clinics used the same set of implementation strategies, and so we were unable to discern which strategies were most impactful. Similarly, because all clinics used the TAPS Tool for screening, we could not test the hypothesis that the screening tool was partially responsible for the higher prevalence of drug use detected here, in comparison to the parent study. The only measure of provider counseling was documentation in the study-provided CDSS, and it is possible that PCPs were having discussions of screening results without documenting it there. Similarly, treatment referrals were only tracked if they were made using the CDSS, and informal referrals or those made by other clinic staff, such as behavioral health providers, were not captured. Conducting chart reviews or using natural language processing to analyze clinical notes may have captured this information, but were outside the scope of our study. Finally, as clinics from a single FQHC in northern Maine, the study clinics are not representative of all rural primary care practices. Notably, the predominantly White patient population served by these clinics is not generalizable to the rural population of the U.S.

## Conclusion

By examining the feasibility of implementing EHR-integrated screening in rural FQHCs, this study expands on our prior findings [19], and may provide guidance for other clinics and health systems that are looking to implement alcohol and drug screening. The high screening rate for screening-eligible annual preventive care visits suggests that EHR-integrated screening for substance use during these types of prevention-focused primary care visits is feasible, and the relatively high prevalence of unhealthy alcohol and drug use detected by screening supports its relevance to medical care. Prior studies have demonstrated the challenges of implementing substance use screening and interventions in medical settings [29, 32, 58–61], and barriers to screening may be even more pronounced in rural practices [3, 11, 20, 21]. Our implementation feasibility study demonstrates that high quality screening can be achieved, and integrated into primary care workflows, by leveraging health information technology including the EHR and tablet-based screening approaches. Importantly, since the completion of our study the FQHC has maintained screening at all of the study clinics, and expanded it to additional visit types, which attests to the perceived acceptability and clinical value of the screening approaches that were implemented. The FQHC has also rolled out the screening program to an additional five practices, and plans to use the TAPS Tool system-wide.

Demonstrating the feasibility of systematic substance use screening is particularly important now, given recent updates to the USPSTF screening recommendations [12, 13] and the HEDIS measure for alcohol screening and brief interventions [62], persistent increases in substance use associated with the COVID-19 pandemic [63], and ongoing opioid-related overdose deaths in rural communities [3, 4]. Although further work is needed to develop models and best practices for linking screening to effective interventions for drug use that can be delivered in primary care, this work represents an important step toward addressing the high burden of substance use and associated morbidity in rural adult primary care populations.

## Data Availability

The datasets used in the current study are available from the corresponding author on reasonable request.
